# Current Challenges in Development of a Database of Three-Dimensional Chemical Structures

**DOI:** 10.3389/fbioe.2015.00066

**Published:** 2015-05-26

**Authors:** Miki H. Maeda

**Affiliations:** ^1^Biomolecular Research Unit, National Institute of Agrobiological Sciences, Tsukuba, Japan

**Keywords:** 2D–3D conversion, 3DMET, CLiDE, InChI, canonical SMILES, chemical database, natural products

## Abstract

We are developing a database named 3DMET, a three-dimensional structure database of natural metabolites. There are two major impediments to the creation of 3D chemical structures from a set of planar structure drawings: the limited accuracy of computer programs and insufficient human resources for manual curation. We have tested some 2D–3D converters to convert 2D structure files from external databases. These automatic conversion processes yielded an excessive number of improper conversions. To ascertain the quality of the conversions, we compared IUPAC Chemical Identifier and canonical SMILES notations before and after conversion. Structures whose notations correspond to each other were regarded as a correct conversion in our present work. We found that chiral inversion is the most serious factor during the improper conversion. In the current stage of our database construction, published books or articles have been resources for additions to our database. Chemicals are usually drawn as pictures on the paper. To save human resources, an optical structure reader was introduced. The program was quite useful but some particular errors were observed during our operation. We hope our trials for producing correct 3D structures will help other developers of chemical programs and curators of chemical databases.

## Introduction

A database called 3DMET, a three-dimensional structure database of natural metabolites, is being developed in our laboratory. In the earliest days, 3D structures were converted from two-dimensional (2D) structures of relevant external databases. Currently, new entries to 3DMET are collected from chemical structures from print form. The bottleneck in our project is curation. Because of the shortage of good curators, several trials for automatic operation were evaluated. All trials utilizing known programs indicated that their accuracy is insufficient.

**Figure 1:**
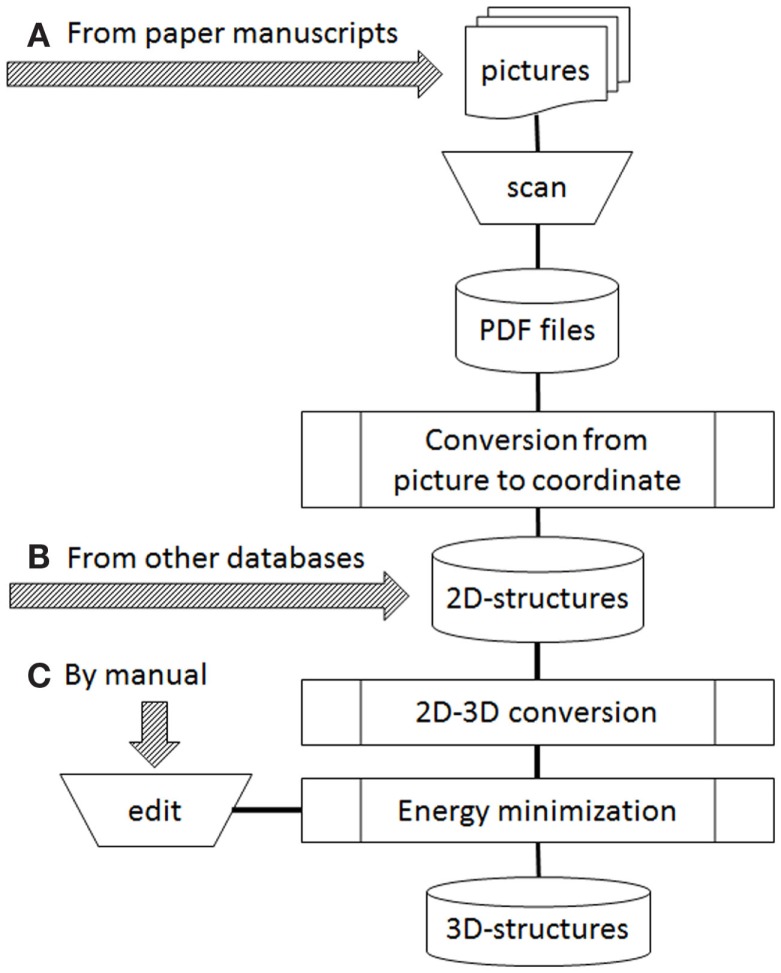
**Operation flow to develop 3D structures**. Arrows of **(A–C)** were possible starting points depended on data sources.

Figure 1 shows our current workflow of 3D-structure construction. During automatic processing, the following three steps are involved: conversion from picture to Cartesian atomic coordinates, 2D–3D conversion, and energy minimization. For conversion from picture to Cartesian coordinates, several programs such as Kekule (McDaniel and Balmuth, 1992), CLiDE (Ibison et al., 1993; CLiDE, 2014), OSRA (Filippov and Nicklaus, 2009), and ChemReader (Park et al., 2009) have been reported. Kekule is one of the earliest programs for optical chemical structure recognition, but it no longer seems to be available. We employed CLiDE supplied by Keymodule Ltd., because it is novel program easily obtainable and easy to handle when we started extracting chemical 2D structures from the paper literature. For 2D–3D conversion, 3D-generators such as CONCORD (Pearlman, 1987), CORINA (Gasteiger et al., 1990; CORINA, 2015), and OMEGA (Bostrom et al., 2003; OMEGA, 2015) were developed. They are widely used programs in drug discovery. We employed CONCORD in the earliest days of our database development. However, we do not use any 3D-generators now because our sources changed from 2D-files to paper literature. Energy minimization programs were necessary in the both cases. We have usually employed MOE (Molecular Operating Environment, 2015) as supplied by Computer Chemistry Group to develop 3D structures using the MMFF94 force field (Halgren, 1996).

For the 3D-structure construction, we initially employed 3D-generators. During the automatic conversion process by 3D-generator and energy minimization, we found excessive amounts of improper conversions (Maeda and Kondo, 2013). To detect improper conversion, we tested several approaches. Finally, we decided to compare IUPAC Chemical Identifier (InChI; Coles et al., 2005; IUPAC Chemical Identifier, 2015) and canonical simplified molecular input line entry specification (SMILES; Weininger, 1988; Weininger et al., 1989; SMILES, 2008) before and after conversion because of convenience and accuracy. The KEGG COMPOUND dataset (Goto et al., 2002) was used to estimate accuracy. Two structures whose InChI or SMILES were the same were defined as correct conversion. All our examined programs made errors, and most of errors were caused by atom chirality or cis/trans inversion. As a result, manual curation was performed to fix the 3D structures.

In our current workflow (Figure 1), three arrows marked as a, b, and c indicate starting points depending on data sources. Two kinds of sources are possible: publicly available databases and printed structures such as in books and journal articles. When the data source is supplied from an external database, it is often provided as 2D structures (all z atomic coordinates are zero in the MDL MOL/SD files). Therefore, to obtain 3D structures, the source data will be operated from Figure 1B. If paper materials are data sources, we follow the path in Figure 1A or Figure 1C. The choice depended on the number of human curators available to work on our project. Based on our experience, we now think that the starting point of manual edit (Figure 1C) is better if enough curators are available. However, when the number of curators is in short supply, we can only choose the starting point of print material (Figure 1A).

Although the accuracies of programs varied in our earlier work (Maeda and Kondo, 2013), nowadays there is no significant difference between available software. We currently employ the program MOE™ 2011 for molecular building because it has an explicit chiral constraint as an option in energy minimization. Some erroneous chirality observed from the former version of MOE no longer occurs in the current version. In this article, we present what we learned from performing construction of the 3D structure of metabolites. We hope that our experience will benefit others working on database curation and will aid future development of relevant software.

## Materials and Methods

### Data sources

KEGG COMPOUND (release 29) was used as a source of 2D structure dataset. This dataset has almost 12,000 compounds. A picture of octadehydro-beta-carotene was taken from “Carotenoids Handbook” edited by Britton et al. (published by Birkhauser). Pictures of samples for chemical literature data extraction were taken from “Insecticides of Natural Origin” edited by Dev and Koul published by Harwood Academic Publishers. Pictures were scanned by Canoscan 9000F (Canon Inc., Tokyo, Japan) and converted to PDF files.

### Conversion from picture to 2D coordinates

Compound pictures scanned from papers were extracted by CLiDE (Ibison et al., 1993) standard 5.2 with default parameters. When large numbers of structures are to be treated, CLiDE professional and CLiDE batch would be more convenient. However, CLiDE standard was taken on trial in this study and pictures were converted one by one. The resolution of input files was set at 400 dpi because of balance of accuracy and operation time during our pre-tests.

### Conversion from 2D structure to 3D structure

2D structure files were transformed to 3D structures by using several versions of MOE. In the following parts, the particular version will be indicated with the result when necessary. Hydrogens were added to the 2D structures, which were then minimized by MOE using the MMFF94x (Halgren, 1996) force field.

### Detection of identity between two structures

IUPAC Chemical Identifier and canonical SMILES were employed to detect identity of 2D and 3D structures. Two structures were converted to InChI and canonical SMILES with chiral options. The two notations were compared in three steps: the initial 2D structure, the structure after addition of hydrogens, and the structures after energy minimization. Chiral tags of phosphate by SMILES are ignored because all oxygen atoms bonding to phosphorus are chemically equivalent. Correspondence between two InChI was estimated as having the same sub-layer strings for c (connectivity), h (hydration), b (double bond), and t (sp3 stereo).

### Calculation of numbers about chiral information

Structures with chiral atoms or bonds were calculated based on InChI or canonical SMILES notation strings. All entries were checked in regard to existence of the t or b sub-layer of InChI and “@,” “∖” or “/” characters of canonical SMILES. Undefined chiral atoms were detected as the “?” character in the t sub-layer of InChI.

## Results

In order to collect chemical structures in a database, it is most basic and effective that skillful and careful chemists handle the data manually (Figure 1C). However, in our team, shortage of chemical curators has been a serious problem since the beginning of this project. In such cases, some steps in the workflow need to be performed automatically by computer or performed by non-chemists, although knowledgeable chemical curators must confirm the structures in the final step of verification. Automation involves two steps: picture to 2D coordinates and 2D–3D conversion processes (refer to Figure 1). All automatic processes must be evaluated for accuracy. Even though energy minimization is not an automated process, it too should be evaluated for accuracy.

### Translation from picture to 2D coordinates

Optical character reader (OCR) is often employed to convert a document from paper to computer readable text files. Similarly, a printed image of a chemical structure can be translated into 2D coordinates as text files. If the converted structures were reliable, 2D structure files could be produced without intervention of chemists. As the result of our investigation, we selected CLiDE distributed by Keymodule Inc.

**Figure 2:**
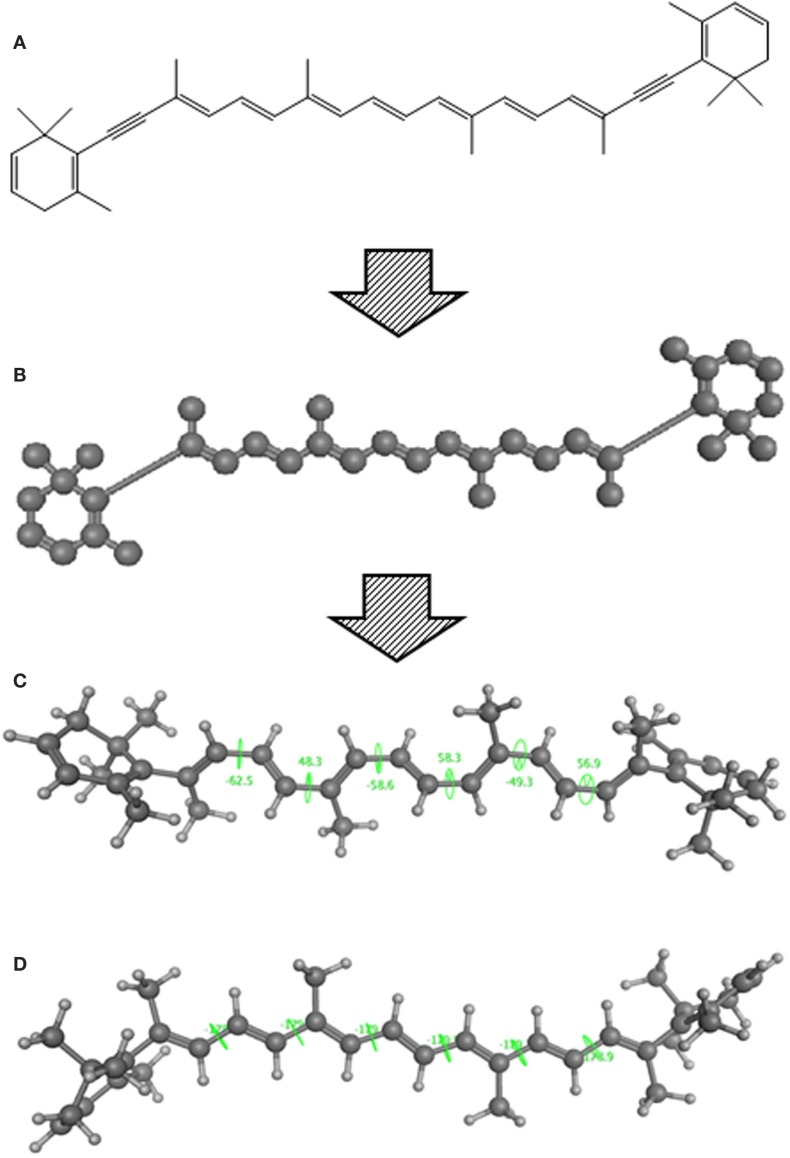
**A schematic example of image to 3D structure about octadehydro-beta-carotene**. Each description shows **(A)** an iniitial structure of 2D drawing, **(B)** the 2D structure after CLiDE, **(C)** the minimized 3D structure with chiral constraint option, and **(D)** directly edited and minimized structure on the MOE window. Green numbers show dihedral angle of the bonds.

Using CLiDE, chemical structures described on paper were translated to MDL mol format files of 2D atomic coordinates. Several types of natural compounds described in books were tested for proper conversion. Figure 2 shows an example of octadehydro-beta-carotene. A printed image of chemical structure (Figure 2A) was converted to a 2D-mol file shown in Figure 2B. Many of the resulting files were correct, but some types of compounds could not be converted well.

Table 1 shows some examples with critical errors: missing characters indicating elements (in examples 3 and 4); missing parts of molecule (in examples 1 and 6); incorrectly recognized chiralities (in example 2); missing cis/trans information (in example 3); and improper recognition of resonance structure (in examples 5 and 6). In some cases, such as example 7, no data were produced.

**Table 1:** **Examples of typical errors for translator**.

	Query	Result	Errors
1. Canavanine	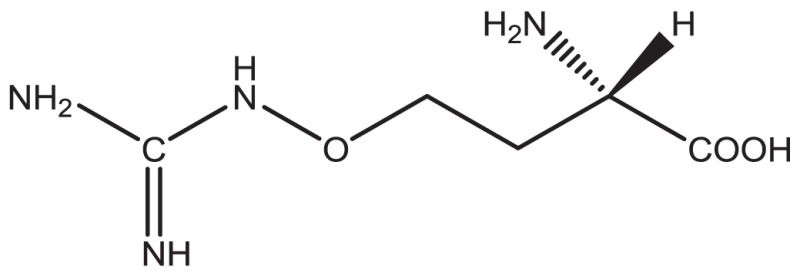	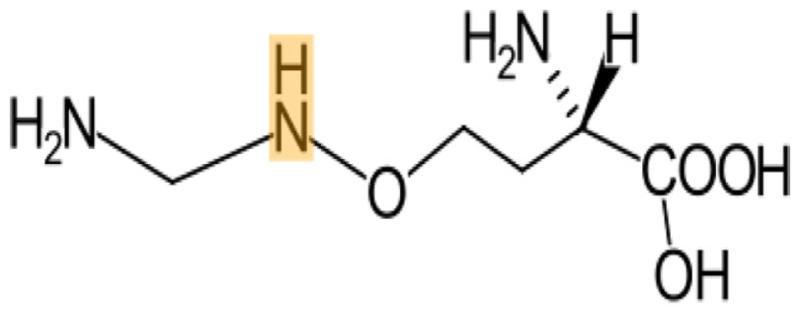	Some elemental descriptions drawn as characters on the paper were not translated
2. Ryanodine	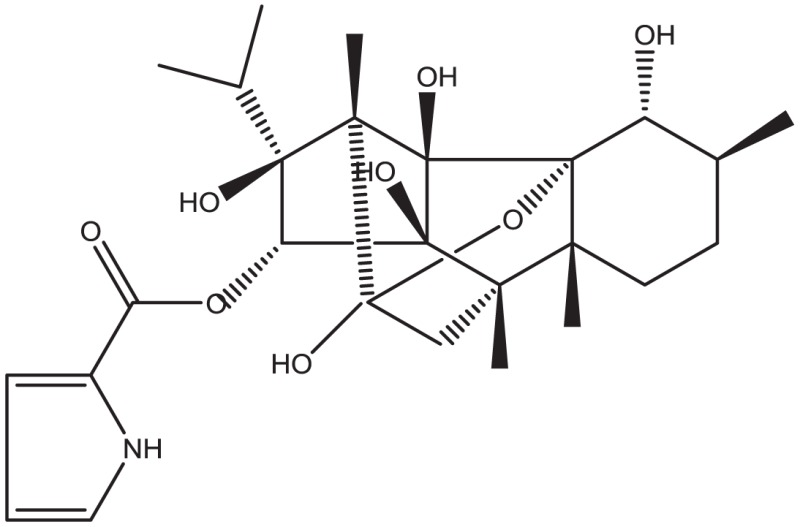	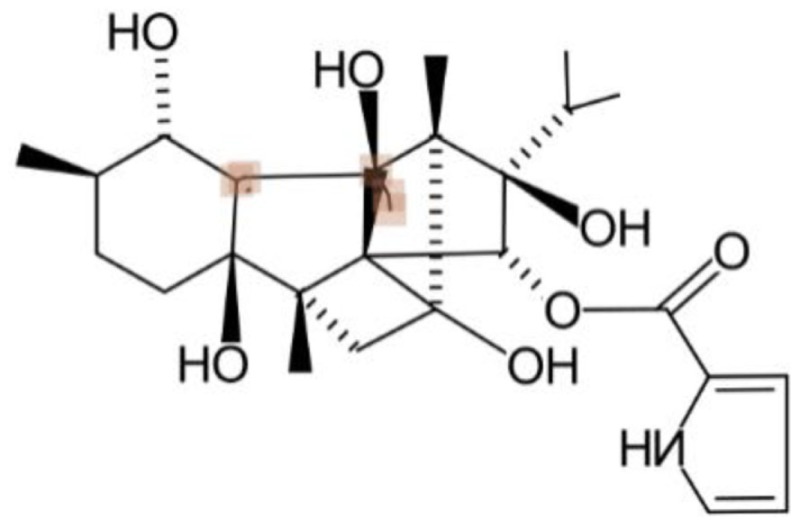	Bond direction and connectivity were not recognized in complex structures
3. Sparteine	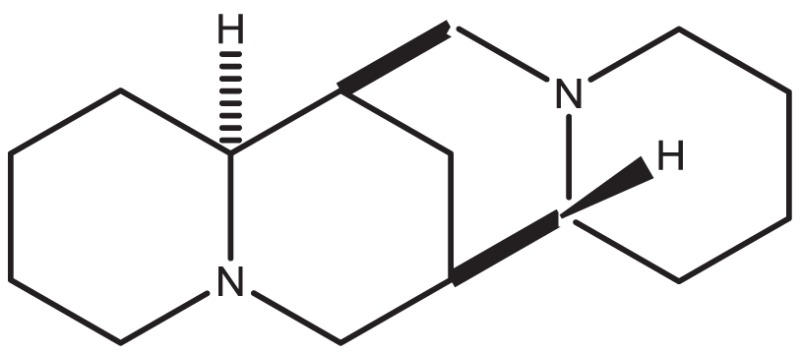	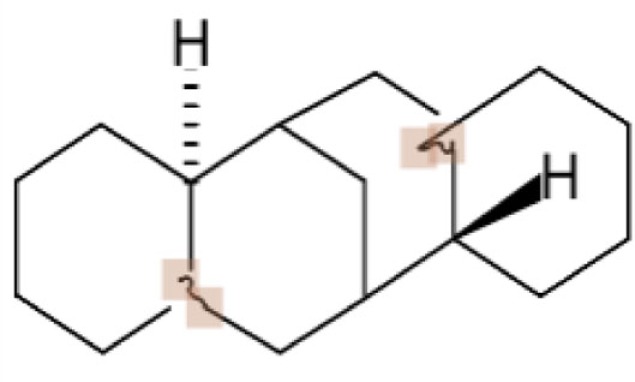	“N” was recognized as a part of bonds. Some kinds of chiral information were lost
4. Terfairine	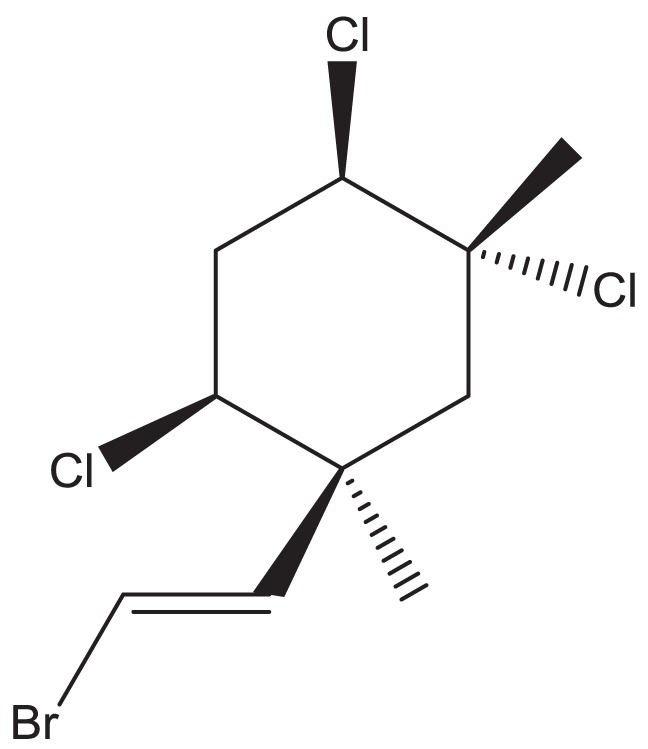	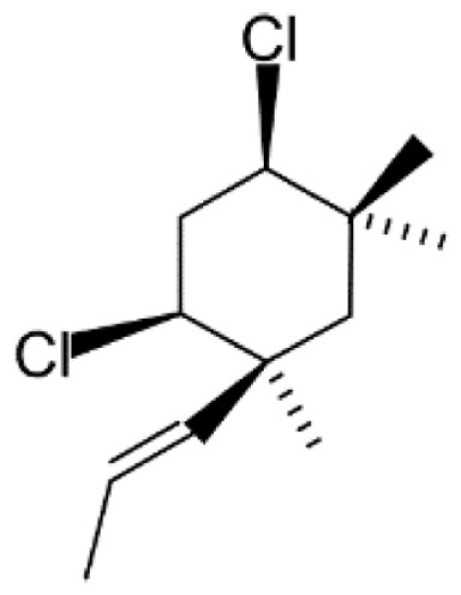	“Br” was not translated as a halogen atom
5. 3(R)-Millonol-B	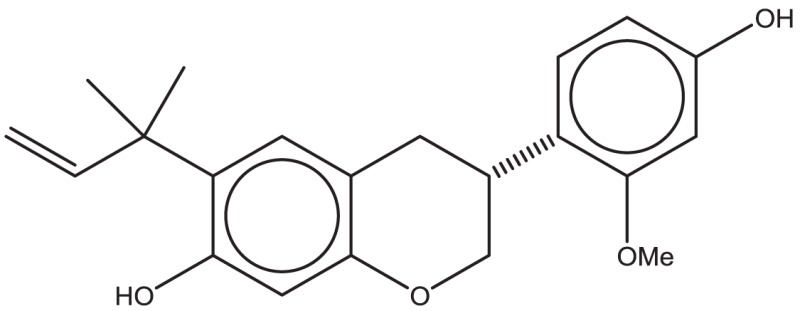	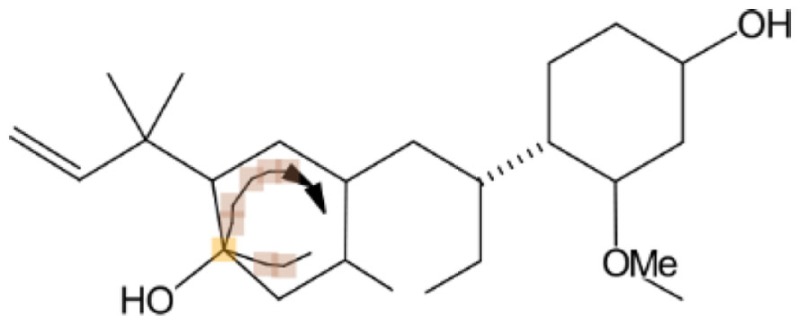	Description of “resonance structure” cannot be converted
6. Veratridine	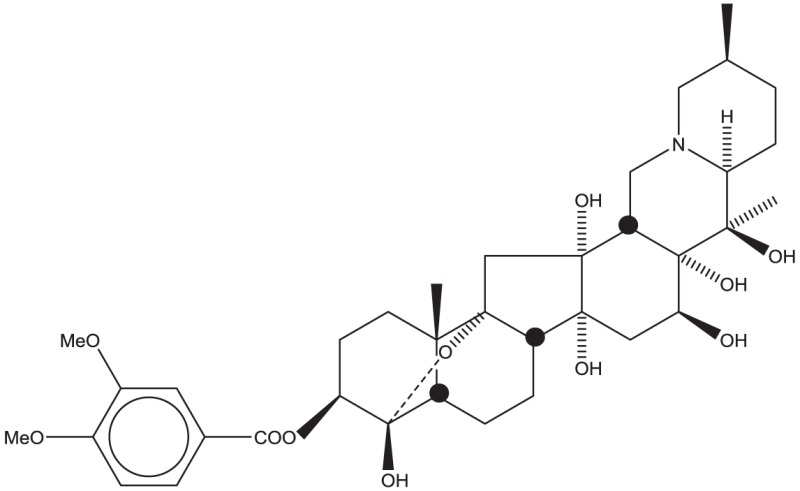	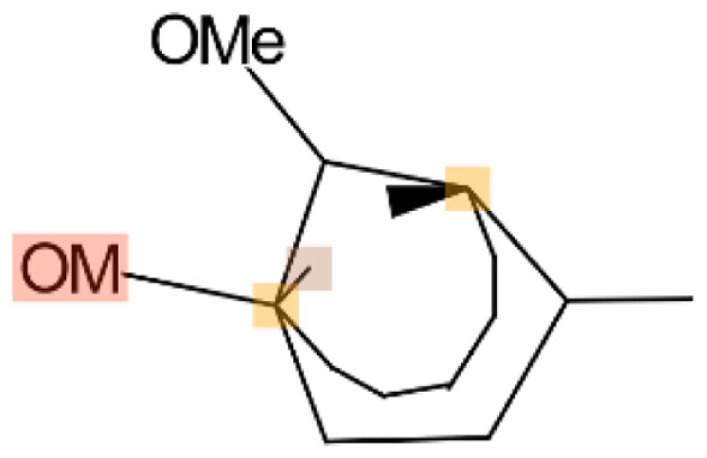	Some parts of the molecule were missing among relatively larger molecules
7. 1,7,9,15-Heptadecatetraene-11,13-diyne	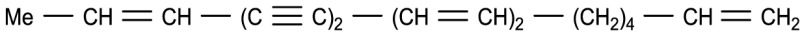	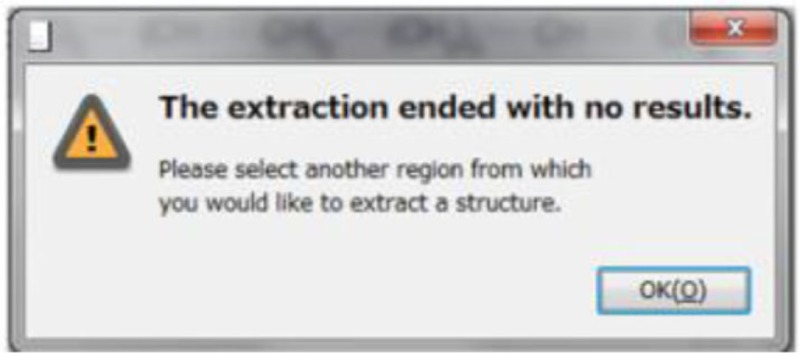	No structures were developed when all atoms were clearly described by elements

*The structures in the “query” column shows 2D-structures of the queries drawn by ChemDraw Pro (2013) version 13 and those in the “result” column are displayed figures from the CLiDE window. Highlighted in orange are possibly improperly translated parts automatically detected*.

### Converting from 2D coordinates to 3D coordinates

Regardless of data source whether scanning from documents on paper or provided molecule files from another database project, it is necessary to convert 2D structure to final 3D structure. In the process, two important problems were encountered, i.e., chiral inversion and quality of the 3D structures. These problems were described in the following two sub-sections, respectively.

#### Detection of Topological Errors

**Figure 3:**
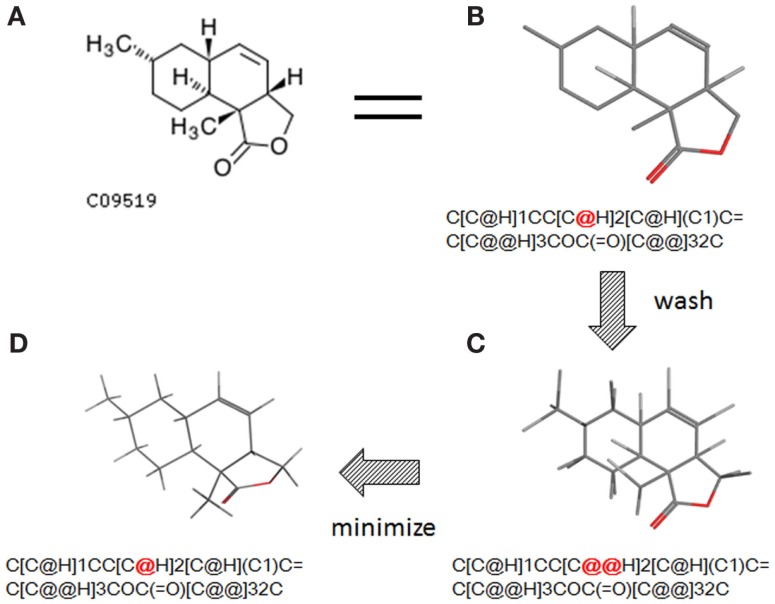
**An example of 2D–3D conversion by MOE**. The C09519 entry of KEGG COMPOUND was converted by MOE. Each description shows **(A)** the molecular drawing provided in KEGG COMPOUND, **(B)** the 2D-mol file viewing by MOE, **(C)** the structure after add hydrogen, and **(D)** the structure after minimization. The structures **(B–D)** are shown with the corresponding SMILES notation strings. Red characters indicate a position of chiral inversion.

The conversion accuracy by MOE 2011 is shown in Table 2. To confirm the retention of chirality in the converted structures, we compared canonical SMILES strings from both 2D and 3D structures, which make the structural evaluation a simple comparison of text strings. Minimizations were performed with default parameters and the MMFF94x force field, and all operations were performed under the chiral constraint option. Most of the chiral information was conserved (because of the chiral constraint). These results were much better than the ones from MOE 2004 or 2005 in our previous report (Maeda and Kondo, 2013). Approximately 4,000 were conserved with MOE 2004 or 2005. The main reason for the chirality inversion is missing chiral tags in the initial 2D files. However, inversion of chirality and cis/trans stereochemistry were still observed. An example of chiral inversion is shown in Figure 3 for KEGG COMPOUND entry C09519. As a result of the whole process, initial 2D structures and 3D structures were correctly converted. However, chiral inversion occurred twice at the same carbon (red characters in canonical SMILES).

**Table 2:** **Frequency of errors regarding chirality and cis/trans stereochemistry during the conversion processes by MOE 2011**.

	cpd – wash	wash – mm	cpd – mm
Same	8,964	11,296	8,735
Chiral inversion	268	119	201
Chiral missing	2,152	521	2,417
Cis/trans inversion	1	29	30
Cis/trans missing	0	6	0
Else unmatched	589	3	621

*All transferred structures were compared, categorized, and counted by each step of database minimization as shown in Table 3. Structures of three steps were compared: the initial COMPOUND 2D-mol files (cpd), structures after wash (wash), and structures after minimization (mm). Each description means as follows: same, completely the same between two SMILES; chiral inversion, atomic chiral tags located on the same position but not the same; chiral missing, at least one of two strings lacking atomic chiral tags; cis/trans inversion, cis/trans tags located on the same position but not the same; cis/trans missing; at least one of two strings lacking cis/trans tags; else unmatched, mismatched by the other reasons*.

IUPAC Chemical Identifier can be also employed to detect correspondence of two structures. Accuracy of two notation strings (canonical SMILES and InChI) was analyzed (Table 3). For the correspondence of whole strings, both led to similar results from the two procedures of command-based generation (6,725 and 6,866 for InChI and SMILES, respectively) and generation by molecular database function (8,522 and 8,735 for InChI and SMILES, respectively). Accuracy for the detection is similar. However, it is clearly different between the structure generation procedures (as SMILES detection, 8,735 by using database function and 6,866 by command-based operation). To determine the reason for results being “different,” stereochemical information in SMILES and in InChI was evaluated. In Table 3, “undefined chiral atoms” means that “?” characters (the undefined chiral tag) were detected in at least one string when compared with InChI or that “@/@@” characters were lost in one string when compared with canonical SMILES. More than 1,000 structures in the original dataset lacked chiral information in the structures. Errors of cis/trans stereochemistry were also observed (about 30 errors were detected by comparison of SMILES). Comparing the numbers of InChI formula layer of Table 3, the correspondence by using command-line operation is better than by using the database function. The major part of this difference between these methods is caused by the salt removal process in the database operation. In our modified KEGG compound dataset, 374 entries consist of two or more molecules.

**Table 3:** **Accuracy of 3D-structure construction by MOE 2011**.

	Command		Database	
	InChI	SMILES	InChI	SMILES
Whole notification strings	11,974	11,974	11,974	11,974
Completely same	6,725	6,866	8,522	8,735
Different	5,249	5,108	3,452	3,239
Chiral errors				
Undefined chiral atoms	1,337	2,513	1,863	2,417
Mismatch about chirality	3,586	2,454	147	201
Mismatch of cis/trans (including undefined bond stereochemistry)	109	33	474	30
Correspondence of InChI layer				
Formula	11,925		9,550	
c (Connection)	11,925		9,569	
h (Hydration)	11,900		9,539	
b (Cis/trans stereochemistry)	11,860		9,217	
t (Chirality)	6,973		7,140	

*Entries of KEGG COMPOUND without “R,” “n,” and “X” were the initial 2D structure dataset (11,974 compounds). Operations of wash and add hydrogen were performed as command-based (command) and molecular database function (database) of MOE. In comparing the initial COMPOUND 2D-mol file and structures after minimization, each description means as follows: same formula, correspondence of formula of main layer in InChI; same string, completely same two strings of InChI or SMILES; chiral difference, unmatched strings with same connectivity and hydration; undefined chiral atom, at least one of two strings lacking atomic chiral information; and chiral mismatch, not corresponding atomic chirality*.

The number of structures with chiral atoms and bonds was also estimated (Table 4). In our initial dataset of KEGG COMPOUND containing 11,974 structures, the entries with atomic chiral tags by InChI (detected as existence of t-sublayer) and canonical SMILES (detected as existence of “@” tags) were 6,608 and 6,704, respectively. The resulting 3D structures with chiral atoms were approximately 8,000 entries. In the same manner, the entries with cis/trans tags by InChI (b-sublayer) and canonical SMILES (“∖” or “/” tags) for initial 2D-files were 1,895 and 1,564, and for final 3D structures were 1,906 and 1,560, respectively.

**Table 4:** **Chiral detection by InChI and canonical SMILES**.

	Chiral tags		Cis/trans tags		Undefined chiral atoms
	InChI	Canonical SMILES	InChI	Canonical SMILES	InChI
Initial 2D structures	6,618	6,704	1,895	1,564	1,316
After wash	8,010	7,803	1,942	1,555	3,045
After minimization	7,268	8,034	1,906	1,560	162

*A number of structures with chiral tags were indicated. Calculation was based on 11,974 compounds from KEGG COMPOUND*.

#### Accuracy of 3D Structure

The next problem we address is the correctness of the whole structure of molecule with accurate partial structures. A structure obtained with correct chirality and cis/trans stereochemistry is shown in Figure 2C. This structure may be chemically erroneous. The first concern is dihedral angles at double bonds. Dihedral angles of single bonds between two double bonds are also shown in Figure 2C. The range of the values is −62.5° to 58.3°. Continuous and intermittent double-bonded structures such as 2,4,6-octa-triene are considered to have a planar conformation because such double bonds are conjugated. The second concern is the cis/trans stereochemistry around double bonds. Even if all double-bonded atoms are located in a plane, double bonds are cis-located about single bonds in between as shown in Figure 2D. Usually, larger substitution is considered far from each other because of steric hindrance. When the single bonds of Figure 2C were set to around 0°, the minimized structure as shown in Figure 2D was obtained. The molecular mechanics energies of structures 2c and 2d were 117.929 kcal and 103.632 kcal, respectively, as calculated by MOE 2011 with the MMFF94x force field. Conformation 2d is of lower energy.

## Discussion

### Hurdle of data exchange between databases

Some chemical databases such as KEGG COMPOUND and ZINC (Irwin et al., 2012) have 2D structures. Developing a database requires extensive human resources. Thus, it would be desirable to share reliable data among databases.

In our case, we adopted an SD file of KEGG COMPOUND because the database is recognized as a standard set of natural products. During the process of our development, we found the dataset contains undefined atoms and/or residues. For example, COMPOUND entry of C00045 is “amino acid” containing one “R” residue (the formula is shown as “C2H4NO2R”). Generally, R of amino acids contained in proteins implies 20 kinds of side chain variation and does not define just one compound. Similarly, “X” and “n” mean some kinds of halogens and *n*-times repeat of a unit, respectively. This kind of description is needed for the purpose of KEGG COMPOUND but is not suitable for our objective. As the result, these entries should be removed because our database requires only defined compound structures.

During the analysis of chiral inversion problem, we found that many entries of external data had no chiral tags of atoms. Because both of 2D–3D converters and “add hydrogens” randomly give a chiral tag to the atom which chiral information is not explicitly given, the resulting 3D structures can contain wrong chiral information. Random addition of chiral tags is permitted to calculate accuracy when there is no chiral definition in pre-converted structures. Most of basic metabolites described in “the metabolic map” should be defined with all chiral information about atoms and bonds. However, not all books describe with complete chiral tags because the chirality may be common knowledge among some chemists. Therefore, we need to verify the new structural data of such compounds.

ZINC and PubChem (Wang et al., 2009) contain a much larger number of natural/artificial compounds than any of the other compound databases. Because of the large number of artificial compounds in such databases, it would be tedious to screen them out from the natural compounds. For this reason, the databases were not chosen as our database resources.

### Limitation on three-dimensionalization from 2D structure

In the process of 3D-structure generation, two main problems occurred. The first problem arises from the nature of the software, and the other is due to the limitation of molecular mechanics calculations.

Accuracy of six 3D generators containing CONCORD and CORINA was compared in the previous report by Sadowski et al. (1994). In the study, 639 X-ray structures in Cambridge Structural Database were analyzed. The dataset contained 213 structures with chiral centers and 35 structures with cis/trans stereochemical double bonds. For all compounds of the dataset, CONCORD and CORINA could generate structures correctly. However, we found that CONCORD makes mistakes in conversion (Maeda and Kondo, 2013). We did not confirm the details of the Sadowski’s set. More complex structures would be encountered by automatic generation of natural compounds such as carotenoids and sugars.

It was also reported that many chiral inversions occurred during three-dimensionalization processes by MOE 2004 or 2005 in our previous report (Maeda and Kondo, 2013). We (and probably many other users also) have requested that the software developers fix the problem. In version 2011, conservation accuracy during minimization is greatly improved with the chiral constrain option. As seen in Table 2, 94.3% (11,296 entries) of the structures with chiral information are correctly handled during the energy minimization process (wash – mm). Erroneous conversion of chirality and cis/trans stereochemistry now total only 1.2% (148 entries), while 4.4% (527 entries) of structures lack stereochemical information and cannot be assessed. Thus, we consider that the new software version is acceptable.

Though the example of C09519 shown in Figure 3 is counted as a correct conversion, chiral inversion is still observed during the two processes of 3D generation and energy minimization. Minimization with the option to conserve chirality in MOE 2011 makes fewer errors regarding atom inversion, and hence we use this version of MOE. However, chiral inversion still occurred. Many of the inversions involved chirality (around 200 structures for database operation). Inversion of cis/trans stereochemistry (E–Z or vice versa) was observed in a few cases (about 30 structures). We usually use command base operation in development of 3D structures. However, chiral inversion occurs much less when using database operation.

The second problem pertains to conjugated double bonds. When a carotenoid structure drawn on the paper was transferred to a 2D-mol file and converted to a 3D structure, a planar conformation of conjugated double bonds could not be obtained. The reason for this problem appears to be caused by inappropriate bond lengths in the initial 2D structures. When we observed the three-dimensionalization process on MOE’s graphic window, the molecule stretched to an extended conformation. The bond length of converted 2D structures from pictures (0.90–0.98 Å) was shorter than ideal (the length of an optimized C–C bond in MOE is about 1.5 Å). This problem can be avoided by manual input. When 3D structures are manually constructed, the conjugated double bond moiety is planar. If such structures were optimized by energy minimization, the structure of continuous double bonds will be put in a planar conformation initially.

The other problem about double bonds concerns connected double bonds as in allenes. Two double bonds of allene are at 90° because of the angle of two pi orbitals. When allenes were edited on the MOE window, two double bonds after MMFF94x minimization were often located in a plane (around 180°). However, some allenes made by the same procedure minimized at around 90°. The difference was not caused by the initial conformation but would be influenced by the other parts of the molecule, for example, as in steric hindrance. If strictly correct structures must be obtained, we could carry out semi-empirical molecular orbital (MO) calculations. In MOE 2011 and later, the PM3 (Stewart, 1989), AM1 (Dewar et al., 1985), or MNDO (Dewar and Thiel, 1977) parameters in MOPAC (Stewart, 1990) can be selected for energy minimization. Generally, the compounds we process are too large for routine use of *ab initio* methods.

### Notes about manual operation

Nowadays, new structures are often made manually for our database 3DMET. The operation is performed by curators. During the process, some problems of 3D structures development were revealed.

One problem is a limitation of molecular mechanics calculation. The MMFF94x force field is used for energy minimization in our current work. This is generally regarded as a relatively reliable and versatile force field. However, structures of allenes cannot be treated properly, as described above. Because molecular mechanics (MM) energy minimization does not adequately consider electronic structure, resulting 3D structures are sometimes not reproduced properly. To avoid this problem, molecular orbital calculations can be adopted if it is necessary to obtain accurate structures. We previously tried to run *ab initio* MO calculations on all structures. However, this required too much time to perform energy minimization and some structures were not optimized because of oscillation. Although strictly correct structures are desired for investigators of molecular docking, some compromises are necessary. For example, docking programs such as FlexX (Rarey et al., 1996) adopt an incremental construction algorithm. If using this type of program, stereochemical information is important but strictly correct conformational information may not be necessary. Therefore, we collect structures according to the following policy. All entries were generated by MM calculation. When curators think that molecular mechanics is inadequate or misleading, they can use quantum chemistry to generate better 3D structures.

The second problem involves manual mistakes. Many persons have served as chemical curators in our 3DMET development group. In our experience, to increase reliability of contents, it is important that curators have enough knowledge of stereochemistry. However, even with the most skillful chemists, manual operation can sometimes lead to mistakes. The frequencies and type of mistakes depended on the person. Thus, in our current project, manually constructed chemical structures are verified by another curator to avoid wrong structures. The next release of 3DMET will be published with curated 3D structures by the above protocol.

### Evaluation of two structures including stereochemical correspondence

In this study, two structures were compared by correspondence between InChI and canonical SMILES. Before a decision to employ InChI and canonical SMILES, we also evaluated other programs. Regarding SMILES, the programs provided with SYBYL (Isomeric SMILES; SYBYL, 2015) and MOE (Unique SMILES) were tested. As mentioned before, a MOE function named “aRSChirality” was also evaluated because chiral inversions were the main mistakes. In this section, the results are summarized.

**Figure 4:**
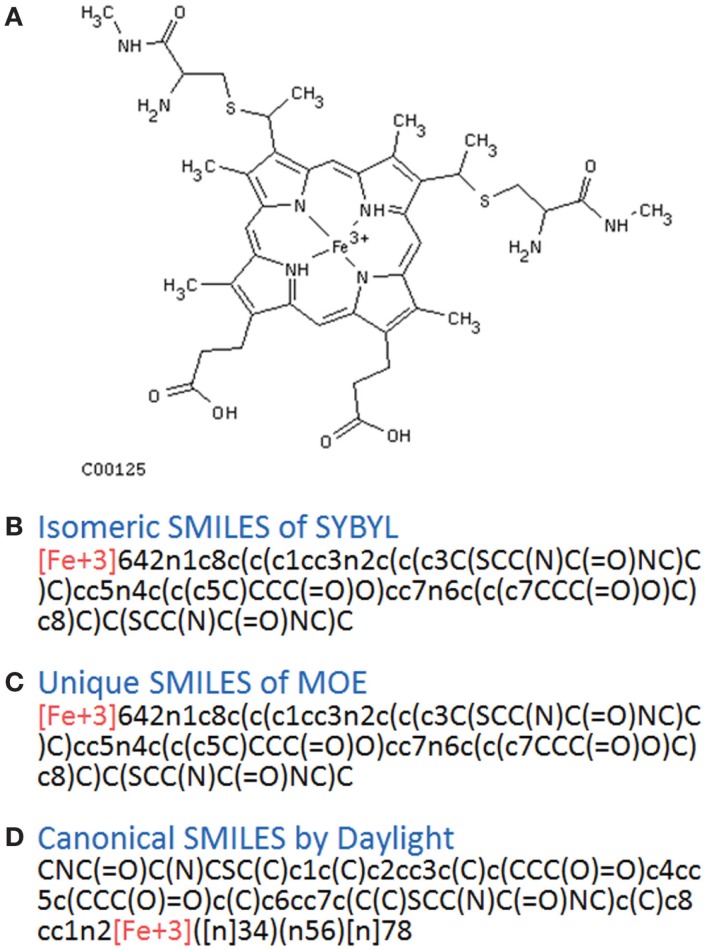
**Several SMILES output**. The C00125 structure of COMPOUND **(A)** was transferred to three SMILES notation: **(B)** isomeric SMILES of SYBYL, **(C)** unique SMILES of MOE, and **(D)** canonical SMILES by Daylight.

In principle, several SMILES notations can be output from one chemical structure depending on the first atom selected. For duplicate detection, one string should mean only one structure. Canonical SMILES, Isomeric SMILES, and Unique SMILES should be all developed to gain a “unique” SMILES notation for such purpose. However, the outputs of unique notations were different, such as in the example of C00125 of COMPOUND shown in Figure 4. The differences were caused by the choice of the first atom. Canonicalization of SMILES by Daylight was explained in the report of the algorithm (Weininger et al., 1989). The first atom is defined as the end of the longest chain in the molecule, whereas in Isomeric SMILES and Unique SMILES the atom with the largest mass is selected as the first atom. Therefore, isomeric SMILES and unique SMILES were the same in the example of Figure 4. At first, we employed isomeric SMILES to detect correspondence of two structures. However, current detection of non-correspondence between two structures is mainly carried out by canonical SMILES because more structures were translatable.

Structures that were unable to be translated into SMILES were also analyzed. For canonical SMILES, such structures included a highly complex portion, especially for symmetric structures. The structure of C00125 (Figure 4) did provide canonical SMILES. However, some porphyrins with short substituted residues attached to the core structure were not translatable. The reason for this is that the canonicalization has failed.

Conservation of stereochemistry during three-dimensionalization detected by InChI and canonical SMILES is shown in Table 3. Differences in accuracy using InChI or canonical SMILES notation strings were negligible (6,725 and 6,866 command operation and 8,522 and 8,735 database operation, respectively). In our experience, transferable structures were different depending on the notation strings. In such cases, we judged structures being processed to be conserved when the strings were the same.

For detection of correspondence, the aRSChirality function of MOE was also tested. It detects chirality for each atom. The aRSChirality strings of chiral tags order depends on the order of the atoms in the input files and contain tags for hydrogens. When two structures are compared by this method, the orders of the atoms should be the same and tags of hydrogens should be removed. Several years ago we judged it best to use canonical SMILES rather than aRSChirality. Recently, an SVL script for MOE was coded to correspond atoms of two structures by the MOE supporting staff of Ryoka Systems Inc. Thus, it may be applicable to check chirality now (we did not estimate detailed results).

### Messages from database developers to program developers

Problems during the generation of chemical structures of 3DMET have been described. Our motivation in this paper is to help make software developers aware of present problems in construction of chemical databases. The tested programs generate adequate structures most of time, but further improvement of the algorithms is desired for better results.

Most of the observed errors involve inversion of chirality. Wrong cis/trans stereochemistry was also observed. However, it is encountered much less than chiral inversion. The errors may be the result of lack of sufficient information on chirality and cis/trans stereochemistry. Bond and atom types are defined in the molecular mechanics programs. Usually, molecular connectivity is described as graphs consisting of atoms (nodes) and bonds (edges). Fundamentally, the direction of edges is not defined in graphs. If no limitation for bonds were programed, chiral information would be ignored during calculation. The recent version of MOE (2011 and later) can limit errors on chiral definition (Table 4). Thus, we use MOE 2011 to make 3D structures in our manual process. In the constructed chemical structures, errors on chirality are still observed. Further improvement is desired for automatic treatment. As a user, we should pay attention if we use 3D-structure datasets that were developed with automation.

## Conflict of Interest Statement

The author declares that the research was conducted in the absence of any commercial or financial relationships that could be construed as a potential conflict of interest.
